# Opening of triangular hole in triangular-shaped chemical vapor deposited hexagonal boron nitride crystal

**DOI:** 10.1038/srep10426

**Published:** 2015-05-21

**Authors:** Subash Sharma, Golap Kalita, Riteshkumar Vishwakarma, Zurita Zulkifli, Masaki Tanemura

**Affiliations:** 1 Department of Frontier Materials, Nagoya Institute of Technology, Gokiso-cho, Showa-ku, Nagoya 466-8555, Japan; 2 Center for Fostering Young and Innovative Researchers, Nagoya Institute of Technology, Gokiso-cho, Showa-ku, Nagoya, 466-8555, Japan

## Abstract

In-plane heterostructure of monolayer hexagonal boron nitride (h-BN) and graphene is of great interest for its tunable bandgap and other unique properties. Here, we reveal a H_2_-induced etching process to introduce triangular hole in triangular-shaped chemical vapor deposited individual h-BN crystal. In this study, we synthesized regular triangular-shaped h-BN crystals with the sizes around 2-10 μm on Cu foil by chemical vapor deposition (CVD). The etching behavior of individual h-BN crystal was investigated by annealing at different temperature in an H_2_:Ar atmosphere. Annealing at 900 °C, etching of h-BN was observed from crystal edges with no visible etching at the center of individual crystals. While, annealing at a temperature ≥950 °C, highly anisotropic etching was observed, where the etched areas were equilateral triangle-shaped with same orientation as that of original h-BN crystal. The etching process and well-defined triangular hole formation can be significant platform to fabricate planar heterostructure with graphene or other two-dimensional (2D) materials.

The discovery of graphene in 2004^1^ opened a wide field of 2D materials for various applications. 2D materials show different electronic and physical properties compared to bulk form due to electron confinement and lack of interlayer interaction^2^. 2D materials are found to be semi-metallic (graphene), insulating (h-BN) and semiconducting (MoS_2_) depending on their electronic band structure^3^,^4^,^5^. h-BN consist of alternating sp^2^ hybridized B and N atoms in a honeycomb structure. It shows interesting properties like very high transparency, thermal conductivity and mechanical strength^6^,^7^. h-BN is also highly oxidation resistant and can remain pristine at a considerably higher temperature^7^,^8^. h-BN is atomically flat with no dangling bond and charge traps, making it an ideal substrate for graphene based electronics without compromising very high carrier mobility of graphene^9^,^10^. It has been observed that a measurable band gap can be induced in graphene using h-BN as substrate, opening a new prospect for Field effect transistor (FET) fabrication^11^,^12^,^13^. Recently, it has been obtained that bandgap can be tuned for graphene/h-BN system under commensurate and incommensurate states^14^. It has been also demonstrated that a hybrid film containing B, N and C atoms can possess a tunable bandgap^15^. Most interestingly, theoretical analysis has predicted that the fundamental properties of h-BN (such as nonmagnetic property) can be altered by embedding graphene in the crystal. Incorporation of graphene will preserve the 2D planar structure of h-BN sheet, as well as unexpected electronic and magnetic properties also can emerge by controlling their shape, size and edge stuctures^16^,^17^,^18^,^19^.

Exfoliation has been used to extract monolayer of h-BN and other 2D materials to investigate the fundamental properties and device applications^20^,^21^,^22^. Recent development shows large-area synthesis of graphene and other high quality 2D materials by a chemical vapor deposition (CVD) process^23^,^24^,^25^,^26^. h-BN films has been synthesized by both atmospheric and low pressure CVD technique^27^,^28^. CVD growth of graphene has been studied extensively than that of h-BN with control over number of layers as well as shape and size of individual crystal^29^,^30^. Recently controllable growth of h-BN on Cu, Ni, Pt, Co and Ru has been significantly explored^31^,^32^,^33^,^34^,^35^. Different factors like growth and annealing temperature, gas flow and choice of precursor can affect the morphology and layer of synthesized h-BN. In graphene related studies by CVD, it was observed that H_2_ played double role as nucleating agent and etching agent^36^,^37^. Again, Ma *et al.* have demonstrated edge-controlled growth and kinetics of single-crystal graphene domains in CVD process^38^. Sutter *et al.* reported H_2_ induced etching of h-BN grown on Ru/sapphire substrate^39^. Most recently, graphene/h-BN hybrid structures have been synthesized by etching graphene and subsequent growth of h-BN on the etched holes^40^. Similarly, Liu *et al.* demonstrated synthesis of h-BN and subsequently graphene around the crystal to create a planer heterostructure^41^. However, etching of individual h-BN crystal with controlled shape and size is still to be explored, which can be significant to develop planar heterostructure to realize exciting electronic and magnetic properties. Hence, exploring the etching process of ordered individual h-BN crystals can be very significant. In contrast to previous findings, we demonstrate for first time possibility of opening triangular hole in individual triangular-shaped h-BN crystals, which will enable to grow graphene and other 2D materials in the etched hole to fabricate a well-defined heterostructure.

## Results and discussion

**Figure 1:**
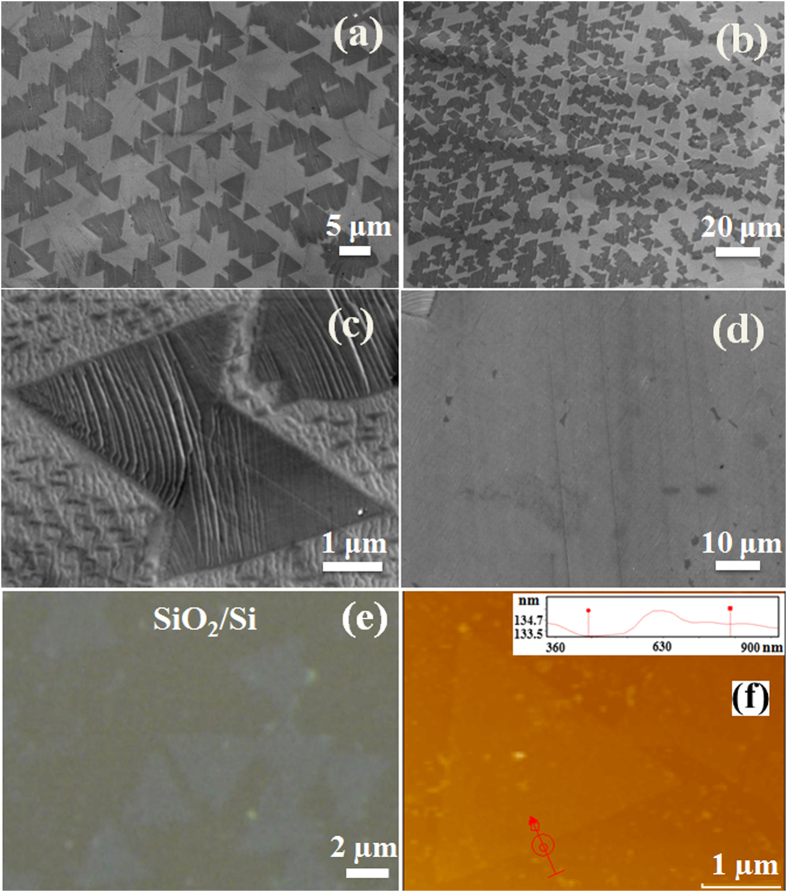
SEM image of (**a**) uniformly distributed triangular-shaped h-BN crystals (**b**) large number of nucleation around cold rolled lines of Cu foil (**c**) triangular h-BN crystals with two possible orientations (**d**) a continuous h-BN film synthesized with growth time of 30 min. (**e**) Optical microscope and (**f**) AFM images of triangular-shaped h-BN crystals transferring on a SiO_2_/Si substrate.

Figure 1(a) shows scanning electron microscope (SEM) images of the uniformly grown triangular h-BN crystals on the Cu substrate. Large number of crystal nucleation can be seen on Cu rolled lines as shown in fig. 1(b). Rough surfaces like cold rolled lines and crystal imperfection such as, edges and defects minimize the activation energy for nucleation of large number of overlapped h-BN crystals (supplementary information, figure S1). In this aspect, high temperature annealing in H_2_ atmosphere minimizes the lines and imperfections by recrystallization of the Cu surface. Figure 1(c) shows a higher resolution image of two different triangular-shaped crystal with opposite orientation, where the two adjacent edges make 120°. Again, theoretical studies have shown that triangular h-BN crystal can consist of only with N terminated edge in zigzag direction^42^,^43^ as it is more favorable energetically compared to other configurations. Triangular h-BN crystals with slightly curved vertices were also observed at some places (supplementary information, figure S2). These types of crystals consist of edges with both B and N atoms^42^. Increasing the growth time around 30 min, individual h-BN crystals ultimately merge together to form a continuous film as shown in fig. 1(d). Figure 1(e) shows an optical microscope image of the transferred triangular h-BN crystals without structural deformation. Thickness of the synthesized h-BN was estimated by atomic force microscope (AFM) studies. Figure 1(f) shows an AFM image of the synthesized h-BN crystal after transferring to SiO_2_/Si substrate, presenting an approximate thickness of around 1.3 nm. However, thickness of all the triangular h-BN crystal is not uniform as observed by the AFM analysis.

**Figure 2:**
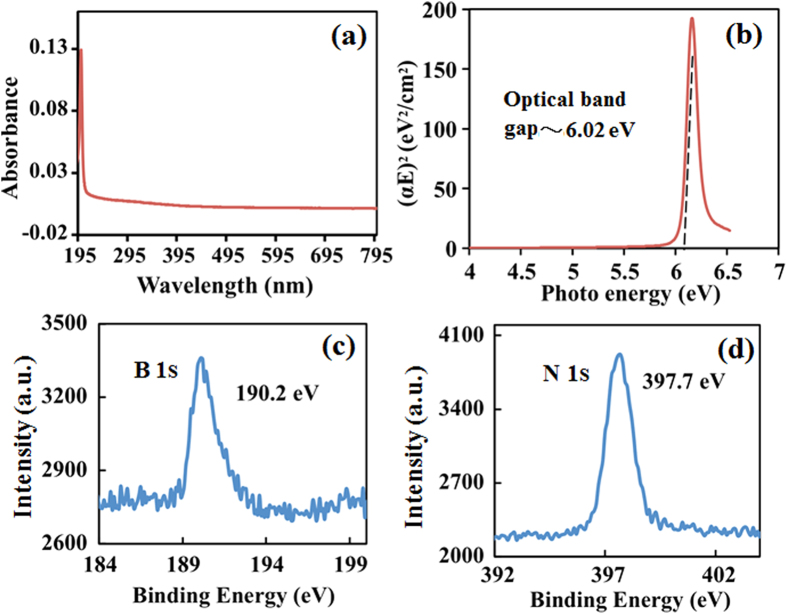
(**a**) UV-visible absorption spectrum and (**b**) Tauc plot of a transferred h-BN film to determine the optical bandgap. (**c**) B 1s and (**d**) N 1s XPS spectra of as-synthesized h-BN, presenting the peak-centered at 190.2 eV and 397.7 eV, respectively.

Figure 2(a) shows UV-visible absorption spectra of an h-BN continuous film after transferring to quartz substrate. The h-BN film is highly transparent from visible to infra-red (IR) wavelength. Further, optical band gap was calculated with a Tauc plot as shows in fig. 2(b). Optical band gap was found to be around 6.02 eV close to the previous reported value for CVD synthesized h-BN^31^. Elemental analysis was performed to measure B and N ratio in the synthesized h-BN using X-ray photoelectron spectroscopy (XPS). Figure 2(c) shows XPS B1s core level spectra with a peak center at 190.2 eV, corresponding to B binding energy. Similarly, fig. 2(d) shows XPS N1s core level spectra with a peak center at 397.7 eV, corresponding to N binding energy. B and N atoms were found to be almost in 1:1 ratio as measured by quantitative analysis of XPS spectra. These results evidently confirm growth of high quality h-BN crystals and continuous film on Cu foil using borazane by our optimized APCVD process. The high quality of the synthesized h-BN crystal is critical to obtain further information in regards of chemical and physical properties. In graphene and h-BN crystal growth process, it has been observed that H_2_ plays a significant role in nucleation as well as large-area growth. In this prospect, we explore the effect of H_2_ in a post growth process.

**Figure 3:**
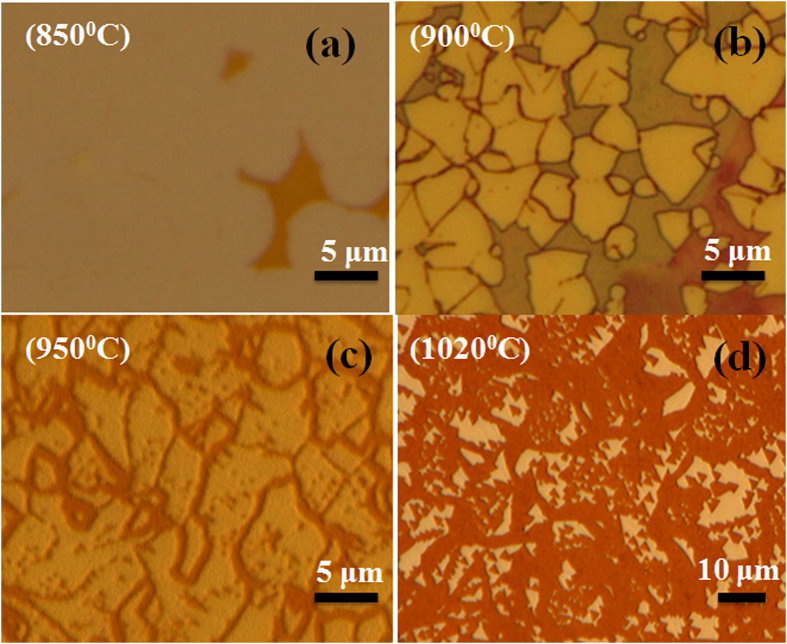
Optical microscopy images of h-BN etched at different annealing temperature (**a**) 850 °C (**b**) 900 °C (**c**) 950 °C and (**d**) 1020 °C.

In case of graphene synthesized on Cu foil, hexagonal etched holes by H_2_ have been observed with etching towards zigzag direction^40^. However, in case of h-BN, etching with edge orientation and preference to particular direction has not previously observed for individual crystals. In this prospect, this is the first report investigating etching of h-BN crystals and continuous film under H_2_ atmosphere at different temperatures. Controlled etching process of h-BN can be significant to create planar hybrid structure with other 2D materials as well as synthesis of h-BN nanoribbons. Etching of h-BN film was carried out with a gas mixture of H_2_ (2 sccm) and Ar (85 sccm) in a post growth process. It is to be noted that we do not observe etching of the h-BN in only Ar atmosphere. Figure 3(a) shows an optical microscope image of h-BN film on Cu foil annealed at 850 °C for 7 min. We can observe some void (Cu with no h-BN film) as shown in the figure to identify the as-grown h-BN on Cu surface. In this case, we did not observe any etching effect of the h-BN film. Subsequently, increasing the annealing temperature to 900 °C, etching of h-BN was observed from crystal edges with no visible etching in center of individual crystal as shown in fig. 3(b). Now, increasing the annealing temperature to 950 °C, formation of etched triangular holes as well as etching from edges can be observed (fig. 3(c)). Further increasing the temperature to 1020 °C, we observed significant amount etching (fig. 3(d)). Interestingly, the edge structures of etched h-BN are clearly pronounced with the crystal forming triangular-shaped. From this observation it can be concluded that the etching is enhanced with increase in temperature and certain activation energy needed for etching to initiate. Two different etching mechanisms are observed in the presented results. At a low temperature (≤900 °C) etching mainly starts from common defects like crystal edge and grain boundary and continuously grows along the defective lines ultimately dividing the crystal or creating island of h-BN. Edges of crystals and grain boundaries are defective compared to the middle part, which makes etching from crystal edges dominant in low temperature. Whereas, increasing the temperature more H_2_ can dissolve in Cu surface and H_2_ can move through the Cu substrate and can cause etching upon finding a defective place within a crystal. At temperature ≥950 °C anisotropic etching becomes quite significant forming triangular etched holes in zigzag direction. It was observed that the Cu substrate acted as catalyst in graphene etching mechanism. To study role of catalytic substrate in h-BN etching, h-BN film was transferred to SiO_2_ substrate and subjected to annealing at 1000 °C for 7 min under our standard condition. Anisotropic etching of h-BN was not observed on the SiO_2_ substrate, rather composition of the h-BN was significantly changed (supplementary information, figure S5). This shows the importance of H_2_ interaction with Cu surface for anisotropic etching of h-BN crystals. In the etching process, Cu surface can absorb more and more H_2_ at the etcting initiated sites, which can accelerate the etching reaction.

**Figure 4:**
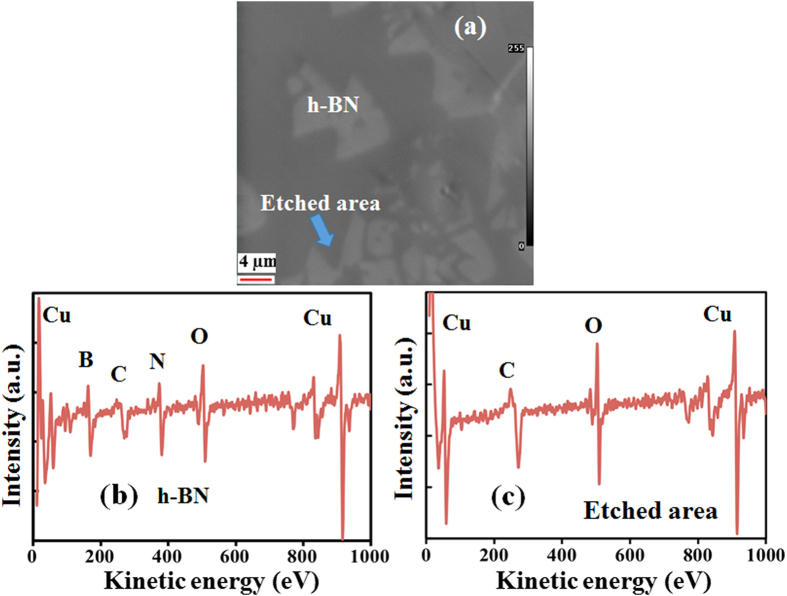
(**a**) SEM image with pronounced etched area of h-BN on Cu surface, elementary analysis were performed by AES analysis at etched and h-BN areas. Auger spectra at (**b**) unetched and (**c**) etched areas of h-BN.

Figure 4(a) shows an SEM image with and without etched areas of h-BN on Cu surface. We performed auger electron spectroscopy (AES) analysis to confirm the etching effect in these samples. Figure 4(b) and 4(c) shows the auger spectra taken at unetched and etched area of h-BN film. B and N peaks were not observed at etched areas. In this case we only observed the C and O peak centered at 247 and 469 eV, along with Cu. The O and C peaks are due to contamination while oxidizing Cu foil in open atmosphere. Whereas, strong B and N peaks with almost equal intensity are observed with peak centers at 171 and 382 eV, along with Cu. Complete absence of B and N atoms indicate clear etching of h-BN. Etching phenomenon is the reverse of growth reaction in which h-BN bonds break and H_2_ can react to form products like NH_3_ and BH_3_. The AES results also evidently confirm the etching process of h-BN crystal synthesized on Cu foil.

**Figure 5:**
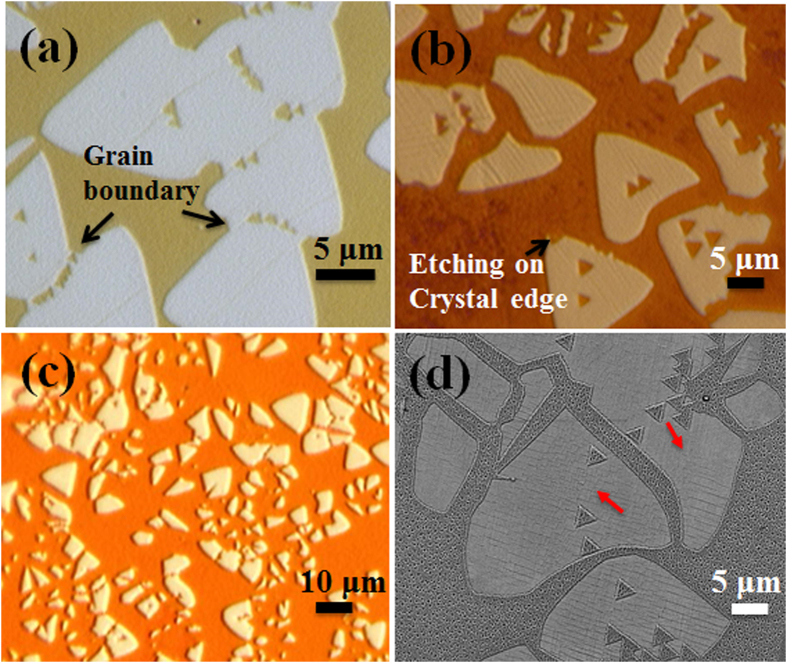
Etching of h-BN crystals with formation of triangular etched holes on (**a**) grain boundary and (**b**) crystal edge. (**c**) Highly etched h-BN with pronounced edge structure and triangular shaped crystal formation. (**d**) Identical orientations of etched triangle within a crystal indicated by red arrow marks.

The interesting anisotropic etching of h-BN with particular shape and orientation was further investigated in details. Figure 5(a) shows interconnected triangular-shaped h-BN crystals after annealing at 1000 °C for 7 min. Etching starts from the crystal imperfections like grain boundaries, crystal edges or from defects (supplementary information, figure S3). Significantly, the etching occurs in triangular shape at the grain or grain boundaries in the zigzag direction. Similarly, fig. 5(b) shows individual triangular-shaped crystals with same shape and orientation with that of etched holes. In this particular temperature range, we observed etching with well-defined edge structure or orientation corresponding to an anisotropic etching process. The etched area continuously increases with increase in annealing duration and ultimately erasing the whole crystal. Figure 5(c) shows etching on a large scale. This is a significant observation in our experiments to control the shape and size of triangular hole enabling defined heterostructure fabrication. Figure 5(d) shows that the triangular holes within a crystal are formed with identical orientations. The red arrows show orientation of etched triangle in two different h-BN crystals. The triangular holes in the same h-BN crystals have same orientation confirming etching in particular direction. The vertices of etched triangular holes are found to be parallel with vertices of the triangular h-BN crystal. This interesting etching phenomena can be significant to create well-defined etched hole and growing other 2D structure such as graphene and thereby realizing in-plane heterostructure. Moreover, controlled etching of continuous h-BN film with ordered structures can be feasible to develop kagome like lattices for spintronic applications^44^.

**Figure 6:**
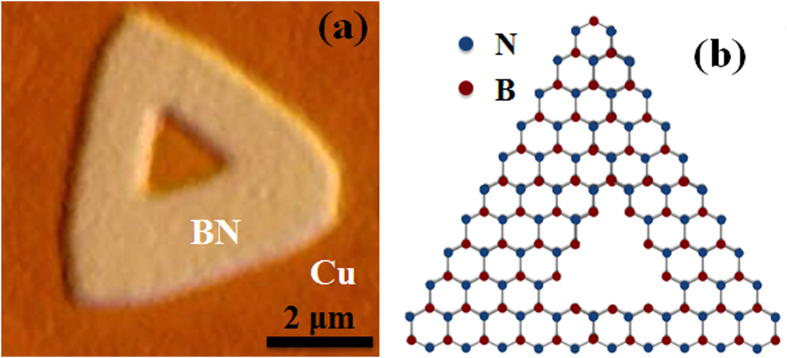
(**a**) Optical microscope image of a triangular shaped h-BN crystal with well-defined edge structure of etched triangular hole at the center. (**b**) Schematic representation of atomic structure of a triangular h-BN crystal with zigzag N-terminated edges.

Recent theoretical studies of h-BN suggest that the edges of triangular-shaped h-BN crystals are N-terminated in Zigzag directions, which is energetically favorable than that of B termination^42^,^43^. According to the theoretical prediction and demonstration, we can analyze the edge structures of well-defined hole created in the h-BN triangle with identical orientation. Figure 6(a) shows an optical microscope image of the triangular-shaped h-BN crystal, where a triangular hole is formed at the center by H_2_-induced etching process. Figure 6(b) shows possible schematic diagram of the h-BN crystal considering the outer most zigzag edges with N-termination. Schematic of other etched structures and hole formation in N-terminated triangular crystal is presented in supplementary information (figure S4). Considering the geometrical arrangement of N-terminated triangular h-BN crystal, inner edges of equilateral triangular hole with identical orientation can be B-terminated. Whereas, the inner edges of equilateral triangular hole with opposite orientation can be N-terminated (supplementary information, figure S4)^45^. In these experiments, we only observed triangular etched hole with identical edge orientation as that of original h-BN crystal. This formation of triangular hole with zigzag edges at the center of the crystal can be significant in terms electronic and physical properties, which is still to be explored. Growth of triangular graphene at the hole of the h-BN crystal can provide us a way to tune electronic, magnetic properties and spin polarization effect. In the etching process, Cu can play as catalyst for H_2_ to react with N and B atoms at surface defects of h-BN crystals. After attaining certain amount of activation energy, B-N bond would break with possible formation of hydrated BH_x_ and NH_x_. A well-ordered triangular hole can form with atom-by-atom removal process from the h-BN crystal. Our findings suggest that Cu-catalyzed H_2_-induced efficient anisotropic etching can be achieved in particular conditions, which create triangular hole etching in the zigzag direction.

In conclusion, we have developed a H_2_-induced etching process of CVD synthesized triangular-shaped h-BN crystals on Cu foil. The etching mechanism was isotropic or anisotropic depending on annealing temperature. Annealing at 900 °C, etching of h-BN was observed from crystal edges with no visible etching in center of individual crystal. Etching of the crystal at temperature ≤900 °C can be utilized for partial grain boundaries etching to identify individual h-BN crystals. While, increasing the annealing temperature to ≥950 °C, etching in triangular holes as well as etching from edges was observed. We observed formation of triangular holes with well-defined edge structure and same orientation of as-synthesized h-BN. The etching and formation of triangular hole with well-defined edge structure can be significant in terms electronic and physical properties. The created triangular holes by etching process can be used as template for growth of graphene and other 2D materials as well as a platform to fabricate planar heterostructure, which can lead observation of unexpected electronic and magnetic properties.

## Methods

### Synthesis of h-BN on Cu foil

In this study, ammonia borane (H_3_NBH_3_) also called borazane was used as precursor for h-BN growth. Borazane is solid at room temperature with B and N atoms in 1:1 stoichmetric ratio. Single furnace system was used for h-BN synthesis. 7 mg of Borazane was taken in a magnetic boat and kept away from the furnace in a horizontal quartz tube during annealing. Cu foil was heated to temperature of 1020 °C and annealed for 30 min in H_2_ atmosphere. Triangular h-BN crystal growth was obtained with a gas mixture of H_2_ (2 sccm) and Ar (85 sccm). The temperature of precursor was gradually increased and stabilized around 130 °C. Borazane decomposes to produce borazine and polymeric amino borane during thermal treatment as confirmed by gas chromatography. Among decomposed products borazine can easily form h-BN by dehydrogenation on Cu substrate whereas polymeric aminoborane can remain as black spots on the Cu substrate. It was observed that maintaining minimum gas flow rate and maximum distance to the growth substrate from precursor boat was essential to get rid of impurities related to aminoborane.

### Hydrogen induced etching process

H_2_-induced etching of triangular crystals and continuous h-BN film at different annealing temperature (850, 900, 950 and 1020 °C) was investigated. In the etching process, precursor flow was stopped after completing the growth of h-BN and annealed in H_2_ and Ar gas mixture varying the temperature.

### Characterization of as-grown and etched h-BN crystals

h-BN crystals synthesized on Cu foil before and after H_2_ etching was analyzed by optical microscopy analysis. Optical microscopy studies were carried out with VHX-500 digital microscope. Field emission SEM analysis was carried out by JEOL JSM-7800F. SEM analysis was also carried out by ultra-high resolution Elionix ESM-9000 to identify the h-BN crystals. AFM study was also carried of the triangular h-BN crystal by JSPM-5200 scanning probe microscope. UV-visible absorption spectroscopy was carried out with JASCO V-670K spectrophotometer. X-ray photoelectron spectroscopy (XPS) analysis was performed by VersaProbe using monochromated Al Kα excitation source (1486.6 eV). Further, elementary analysis of h-BN crystals were carried out with AES in JAMP-7800 Auger microscope.

## Additional Information

**How to cite this article**: Sharma, S. *et al.* Opening of triangular hole in triangular-shaped chemical vapor deposited hexagonal boron nitride crystal. *Sci. Rep.* **5**, 10426; doi: 10.1038/srep10426 (2015).

## Supplementary Material

Supplementary Information
