# Supplementary material for: Opening of triangular hole in triangular-shaped chemical vapor deposited hexagonal boron nitride crystal
Source: Sci Rep. 2015 May 21;5:10426. doi: 10.1038/srep10426 (PMC4650756; doi:10.1038/srep10426)
Supplement: Supplementary Information [file srep10426-s1.pdf]

Supporting Information

Opening of triangular hole in triangular-shaped chemical vapor deposited hexagonal boron nitride crystal

Subash Sharma[*1], *Golap Kalita* [*1 2], *Riteshkumar Vishwakarma*[1], *Zurita Zulkifli*[1], *Masaki Tanemura*[1]

[1]Department of Frontier Materials, Nagoya Institute of Technology, Gokiso-cho, Showa-ku, Nagoya 466-8555, Japan

[2]Center for Fostering Young and Innovative Researchers, Nagoya Institute of Technology, Gokiso-cho, Showa-ku, Nagoya, 466-8555, Japan

Corresponding author: sharmasubash2006@yahoo.com (S.S.) or kalita.golap@nitech.ac.jp (G.K.)

(1) Experimental methods

1.1 h-BN growth method. h-BN film was synthesized on Cu foil (25 μm, Nilaco corp.) by atmospheric pressure chemical vapor deposition (APCVD). Our CVD system consists of a single split furnace with horizontal quartz tube (length 90 cm and diameter 4 cm) as CVD chamber. Cu foil was sonicated in acetone for 30 min. to get rid of impurities and subsequently inserted in the CVD chamber after drying. Cu foil was annealed at 1020^0 C for 30 min. in H_2 atmosphere before the growth. After annealing, gas composition was changed to a mixture of Ar (85 sccm) and H_2 (2 sccm). For the h-BN synthesis, ammonia borane was initially kept inside the quartz tube on a magnetic boat far from the high temperature zone. When, the annealing of Cu foil was completed, the magnetic boat was moved toward the growth furnace. Ammonia borane starts to sublimate and evaporate with increase in temperature near the hot furnace. Growth period usually last from 5 min to 30 min depending upon film requirement (number of layers, crystal size, film continuity etc.). Rate of evaporation of precursor can be controlled by changing distance between precursor boat and furnace. After growth was completed the high temperature furnace was cooled down to room temperature without changing the gas mixture composition and flow rate.

1.2 h-BN transfer method. In the transfer process, a thin layer of polymethyl methacrylate (PMMA) was spin coated on h-BN/Cu and dried for 1 hour in atmospheric condition. PMMA coated h-BN/Cu was put in $Fe(NO_3)_3$ solution for etching. After complete etching of Cu substrate etchant was removed and PMMA/h-BN subjected to several cycles of washing with deionized water and treating with dilute HNO_3 to remove iron residues. Cleaned PMMA/h-BN was transferred to desired substrate (TEM grid or quartz) and dried

for 30 min. After well attachment of the PMMA/h-BN layer on desired substrate, PMMA layer was removed by treating in hot acetone.

(2) Characterization of h-BN crystals

2.1 Morphology of h-BN crystal on rough Cu surface. Figure S1 (a) and (b) shows SEM and optical microscope images of h-BN on Cu terraces and twin boundaries. Rough surfaces like cold rolled lines and crystal imperfection such as, edges and defects can minimize the activation energy for h-BN nucleation. Thus, large number of overlapped h-BN crystals were obtained on the terraces and twin boundaries.

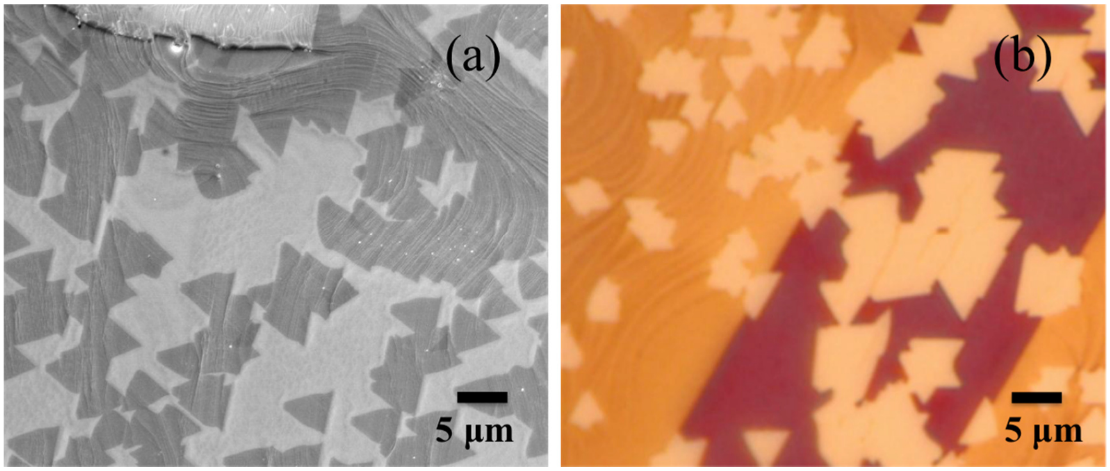

Figure S1 (a) SEM image of h-BN on rough Cu surfaces and terraces (b) optical microscope image of triangular h-BN crystal on grain and twin boundaries.

2.2 Formation of Reuleaux triangular h-BN crystals. We observed formation of Reuleaux triangular h-BN crystals and their interconnected structure as shown in the SEM images (figure S2 (a) and (b)). The polycrystalline Cu foil can have different crystalline orientation, which can affect the structural morphology of the crystal. It has been observed that the reuleaux triangles have edges terminated with both N and B atoms.[1-3]

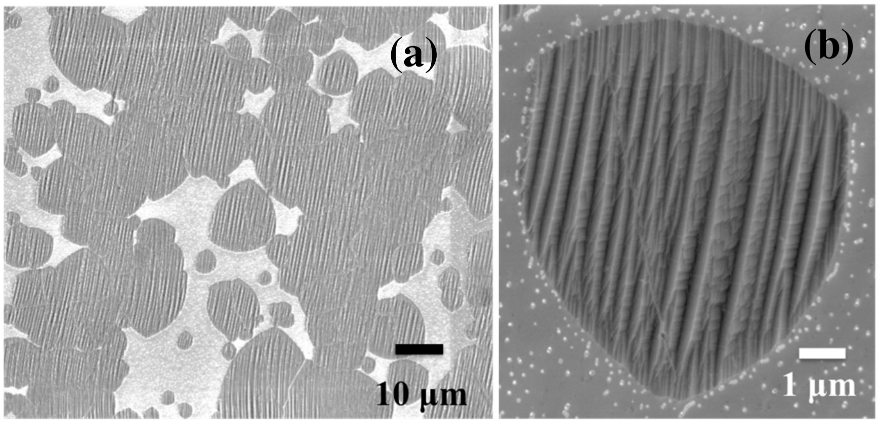

Figure S2 (a) SEM image of (a) interconnected (b) an individual h-BN crystals in the form of reuleaux triangle. The edges of reuleaux triangles are terminated with both N and B atoms.

2.3 Etching of h-BN crystals on Cu foil. Figure S3 (a) shows optical microscope studies of h-BN crystals after the annealing treatment in H_2:Ar atmosphere. The etching of h-BN occurred in triangular shape and particular direction within a crystal. Figure S3 (b) shows an optical microscope image of etching in grain boundaries of two interconnected triangular h-BN crystals.

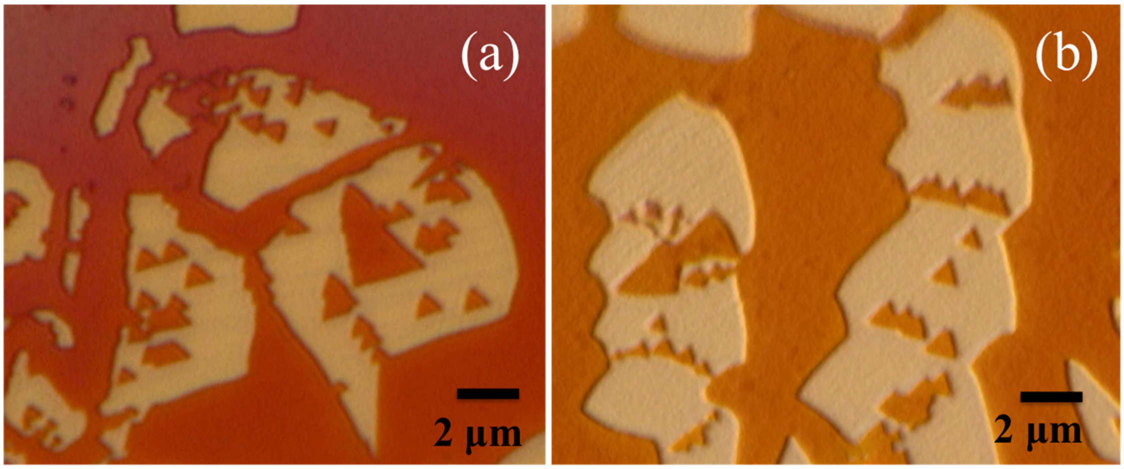

Figure S3 (a) Optical microscope image of etched triangular shaped h-BN with particular direction within a crystal. (b) Etching in grain boundaries of two interconnected crystals.

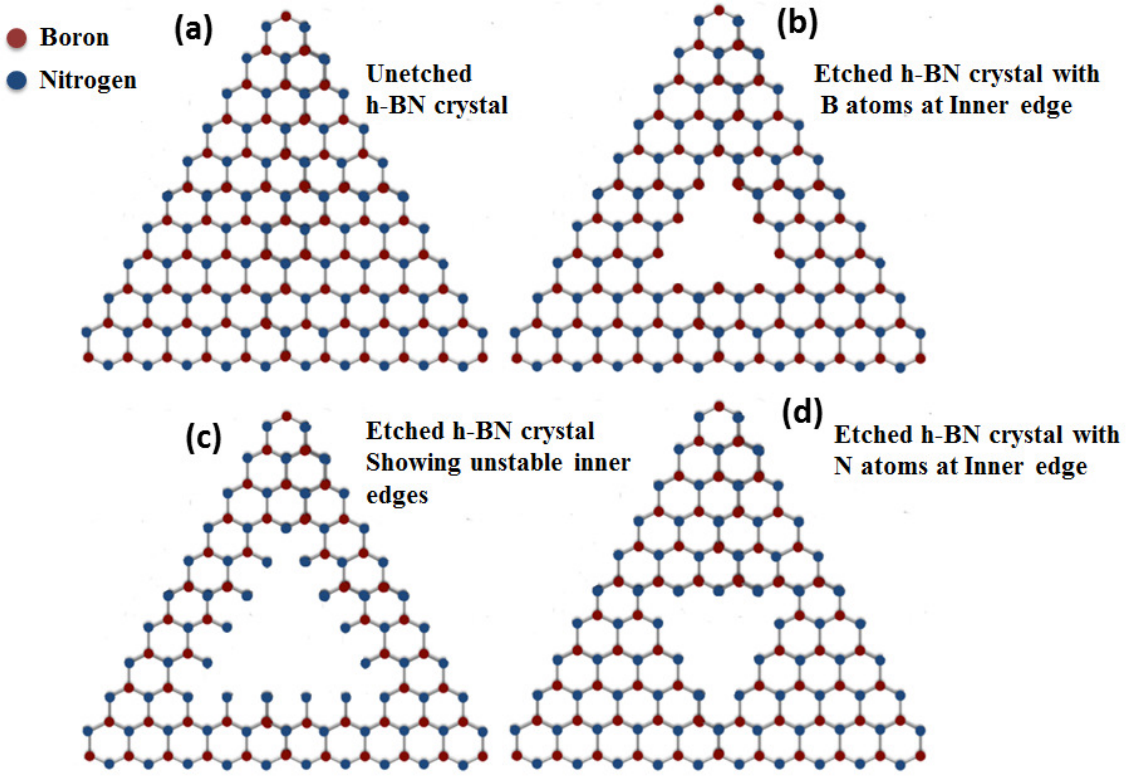

Figure S4 Reconstructed possible structure of the N-terminated triangular-shaped h-BN crystals and formation of triangular etched hole.

Figure S4 shows reconstructed possible structure of the N-terminated triangular-shaped h-BN crystals and formation of triangular etched hole. According previous reports the outer edge of triangular-shaped h-BN crystal is N terminated as presented in figure S5 (a).[3,4] Now, we reconstruct the geometry of triangular hole inside the crystals, considering the three outer most edges are N-terminated. In case of a unilateral triangular hole formation in the triangular-shaped h-BN with same orientation, B-terminated edge can be most favorable (figure S4 (b)). In case of N-termination for same edge orientated hole, it will form an unstable structure with broken hexagons (figure S4 (c)). If, we consider N-termination edge with stable unilateral triangular etched hole structure, the edge orientation

will be completely different (figure S4 (d)) (orientation will be opposite)[5], which we have not observed in the experimental results. Further theoretical and experiment studies are required in these direction, which can provide new information and findings in these direction.

2.3 Etching of h-BN crystals on SiO_2/Si substrate. We have investigated the etching process after transferring to a SiO_2/Si substrate at the same conditions as that of Cu substrate. Figure S5 (a) and (b) shows optical microscope image of transferred h-BN on SiO_2/Si substrate before and after annealing treatment at 1000 ^{0}C.

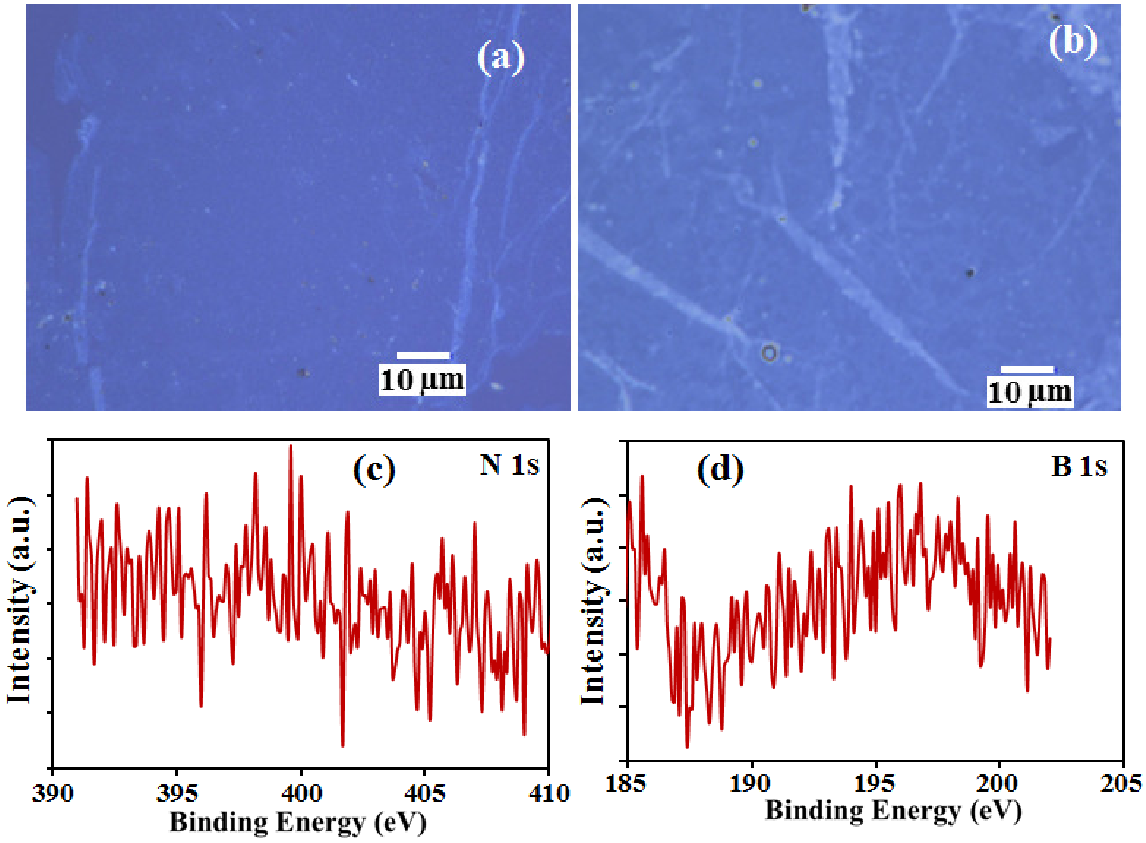

Figure S5 Optical microscope image of transferred h-BN (a) before and (b) after annealing treatment. (c) N1s and (d) B1s XPS spectra of the h-BN on SiO_2/Si substrate after annealing at 1000 ^{0}C.

In this study, we did not observe any anisotropic etching effect of the h-BN on SiO_2/Si substrate. Rather, the composition of the h-BN was significantly changed as observed by the XPS analysis. Figure S5 (c) and (d) shows N 1s and B 1s XPS spectra of the h-BN on SiO_2/Si substrate after annealing at 1000 ^{0}C, which is significantly different than that of original h-BN structure (Figure 2(c) and (d)). This may be due to the reaction with decomposed base SiO_2 layer at such a high temperature (1000 ^{0}C).[6] These results confirm the important catalytic effect of Cu surface in the anisotropic etching process. At a high temperature H_2 can dissolve in Cu surface and move around the crystal through defects inducing etching effect.

References:

(1) Sutter, P.; Lahiri, J.; Albrecht, P.; Sutter, E. Chemical vapor deposition and etching of high-quality monolayer hexagonal boron nitride films. ACS Nano **5**, 7303-7309 (2011).

(2) Liu, L. et al. Heteroepitaxial growth of two-dimensional hexagonal boron nitride templated by graphene edges. Science **343**, 163-167 (2014).

(3) Liu, Y. Bhowmick, S.; Yakobson, B. I. BN white graphene with “Colorful” edges: The energies and morphology. Nano Lett. **11**, 3113-3116 (2011).

(4) Tay, R. Y. et al. Growth of large single-crystalline two-dimensional boron nitride hexagons on electropolished copper. Nano Lett. **14**, 839-846 (2014).

(5) Jin, C.; Lin, F.; Suenaga, K.; Iijima, S. Fabrication of a freestanding boron nitride single layer and its defect assignments. Phys. Rev. Lett. **102**, 195505 (2009).

(6) Bresnehana M. S. et al. Prospects of direct growth boron nitride films as substrates for graphene electronics. J Mater. Res. **29**, 459-471 (2014).
